# Multimodal physiological monitoring in augmented reality teaching environments for children with neurodevelopmental disorders

**DOI:** 10.3389/fnhum.2025.1712662

**Published:** 2026-01-12

**Authors:** Shuyi Zhang, Sukyoung Cho, Fengle Duan, Hao Feng, Qiaoyan Zhang, Muqing Ma

**Affiliations:** 1 School of Humanities and Law, Zhengzhou Shengda University, Zhengzhou, China; 2 College of Education, Sehan University, Yeongam, Republic of Korea

**Keywords:** augmented reality, multimodal sensors, neurodevelopmental disorders, cognitive load, personalized learning

## Abstract

This study investigates the integration of augmented reality (AR) teaching environments with multimodal physiological monitoring for children with neurodevelopmental disorders. We collected EEG, ECG, and eye-tracking data from 115 children (ASD *n* = 45, ADHD *n* = 38, SLD *n* = 32) during AR-enhanced learning tasks. The multimodal fusion approach achieved 89.3% classification accuracy in identifying disorder-specific patterns. Key biomarkers included frontal theta power variations (*p* < 0.001), heart rate variability indices (LF/HF ratio), and fixation duration patterns. AR environments reduced cognitive load by 27% compared to traditional settings while maintaining engagement levels. Personalized intervention based on real-time physiological feedback improved attention performance by 31.2% and social interaction scores by 24.8% over 12 months. These findings demonstrate the efficacy of combining AR technology with physiological monitoring for adaptive special education.

## 1 Introduction

The integration of augmented reality (AR) technology in special education represents a promising approach in addressing the complex learning needs of children with neurodevelopmental disorders. Recent studies demonstrate that AR-enhanced environments can provide structured, engaging, and adaptable learning experiences that accommodate individual cognitive profiles (Tan and Pearce, 2022). The prevalence of neurodevelopmental disorders, including autism spectrum disorder (ASD), attention deficit hyperactivity disorder (ADHD), and specific learning disabilities (SLD), continues to rise, with current estimates indicating that 1 in 68 children are affected by ASD alone (Baio, 2018). Traditional educational approaches often fail to address the heterogeneous nature of these conditions, necessitating innovative technological solutions that can adapt to individual learning patterns and cognitive states.

AR technology offers unique affordances for special education by overlaying digital information onto real-world environments, thereby reducing the cognitive demands of abstract concept formation while maintaining contextual relevance (Mesa-Gresa et al., 2018; Escobedo et al., 2014). The spatial and temporal contiguity provided by AR aligns with cognitive load theory principles, potentially reducing extraneous processing demands and facilitating more efficient learning (Buchner et al., 2022; Suzuki et al., 2024). Studies have shown that AR applications can improve attention, enhance social skills, and support communication development in children with ASD through structured, repeatable practice in controlled environments (Chen et al., 2015; Lorenzo et al., 2019).

The incorporation of physiological monitoring into AR learning environments enables real-time assessment of cognitive and emotional states, providing objective measures of engagement, attention, and cognitive load (Chiossi et al., 2024; Long and Kuhl, 2018). Electroencephalography (EEG) signals reflect neural activity patterns associated with attention and executive function, while electrocardiography (ECG) measures provide insights into autonomic nervous system regulation and stress responses (Berger and Davelaar, 2018; Chicchi Giglioli et al., 2019). Eye-tracking data complement these measures by revealing visual attention patterns and information processing strategies (Pierce et al., 2022).

### 1.1 Literature integration and research gaps

Previous research has established three critical findings that inform our study design. First, AR environments demonstrate cognitive load reduction through spatial contiguity principles (Buchner et al., 2022), though quantification using physiological measures remains limited. Second, individual physiological modalities show promise for neurodevelopmental disorder classification—EEG achieves 70–75% accuracy (Adamou et al., 2020), ECG-based HRV 65–70% (Bellato et al., 2020), and eye-tracking 68–72% —yet multimodal integration has not been systematically evaluated. Third, adaptive learning systems improve educational outcomes by 25–40% (Halkiopoulos and Gkintoni, 2025), but integration with real-time physiological feedback in AR contexts requires empirical validation.

These findings reveal a critical gap: no studies have combined AR technology with multimodal physiological monitoring to create adaptive learning systems for children with neurodevelopmental disorders. This integration is theoretically advantageous because (a) AR reduces cognitive load while maintaining engagement, (b) multimodal physiological data provide complementary information about cognitive and emotional states, and (c) real-time adaptation can optimize learning within each child’s zone of proximal development.

### 1.2 Research hypotheses

Based on the reviewed literature, we propose four primary hypotheses:

*H1 (Cognitive Load Reduction):* AR learning environments will demonstrate significantly reduced cognitive load compared to traditional screen-based instruction, as measured by decreased frontal theta power (>20% reduction), reduced LF/HF ratio (>25% reduction), and more efficient visual scanning patterns (>15% reduction in fixation duration), consistent with spatial contiguity effects observed in previous AR research (Buchner et al., 2022; Suzuki et al., 2024).

*H2 (Multimodal Classification Accuracy):* Multimodal fusion of EEG, ECG, and eye-tracking data will achieve >85% accuracy in classifying neurodevelopmental disorders, representing a > 15% improvement over single-modality approaches, as multimodal integration captures complementary neural, autonomic, and attentional processes (Gramouseni et al., 2023; Kourtesis et al., 2023).

*H3 (Disorder-Specific Biomarkers):* Each neurodevelopmental condition will exhibit distinct physiological signatures: (a) ASD—elevated frontal-temporal gamma coherence during social tasks (Shephard et al., 2018), (b) ADHD—central beta suppression during sustained attention with excessive HRV fluctuation (Adamou et al., 2020; Bellato et al., 2020), and (c) SLD—atypical alpha asymmetry during language processing with increased reading-related regressions (Frazier et al., 2016).

*H4 (Adaptive Learning Efficacy):* Physiologically-informed adaptive AR interventions will produce greater improvements than static AR content across multiple domains, with predicted effect sizes of *d* > 0.8 for attention (based on neurofeedback literature), *d* > 0.6 for social cognition (based on AR social skills training), and *d* > 0.7 for academic skills (based on adaptive learning systems research).

These hypotheses address the fundamental question: Can integrating AR technology with multimodal physiological monitoring create personalized learning environments that objectively assess and adaptively respond to the diverse needs of children with neurodevelopmental disorders?”

### 1.3 Study objectives

To test these hypotheses, the current study pursued three specific objectives:

1. Develop and validate a multimodal classification framework combining EEG, ECG, and eye-tracking data to distinguish between ASD, ADHD, SLD, and typical development during AR learning tasks.
2. Quantify AR environment effects on cognitive load and engagement using objective physiological measures across disorder groups.
3. Implement and evaluate a reinforcement learning-based adaptive intervention system that modifies AR content presentation based on real-time physiological feedback to optimize learning outcomes over 12 months.

## 2 Related work

### 2.1 AR applications in special education

Augmented reality interventions for children with neurodevelopmental disorders have demonstrated efficacy across multiple domains. Studies utilizing AR for social skills training report improvements in emotion recognition, joint attention, and conversational abilities (Lorenzo et al., 2019; Adjorlu et al., 2017). The systematic review by analyzed 47 studies and found that AR applications consistently improved communication outcomes in 78% of interventions for children with ASD. AR-based language learning systems have shown particular promise, with visual augmentation facilitating vocabulary acquisition and semantic understanding in children with language delays (Baragash et al., 2022; Kellems et al., 2025).

### 2.2 Physiological monitoring in educational contexts

The application of physiological sensors in educational settings has evolved from laboratory-based assessments to real-time monitoring systems. EEG-based attention detection algorithms have achieved 85% accuracy in identifying attentional states during learning tasks (Atici-Ulusu et al., 2021). Heart rate variability metrics correlate with cognitive load and emotional regulation, providing continuous assessment of learner states (Valenza et al., 2023; Hadley et al., 2019). Integration of multiple physiological streams through machine learning approaches has improved state detection accuracy by 15–20% compared to single-modality methods (Kourtesis et al., 2023).

### 2.3 Cognitive load assessment in AR environments

Measuring cognitive load in AR contexts presents unique challenges due to the interaction between virtual and physical elements. The NASA-TLX and physiological measures show moderate correlation (*r* = 0.65) in AR learning tasks. Studies comparing AR to traditional instruction report mixed findings regarding cognitive load, with design quality and task complexity moderating outcomes (Akçayır and Akçayır, 2017; Skulmowski and Rey, 2021). Recent work suggests that AR can reduce extraneous load through spatial contiguity while potentially increasing germane load through enhanced engagement (Mayer, 2019; Paas et al., 2020).

### 2.4 Machine learning for multimodal fusion

Advanced machine learning techniques enable effective integration of heterogeneous physiological signals. Deep learning architectures, particularly convolutional neural networks and recurrent models, have shown superior performance in multimodal classification tasks (Paszke et al., 2019). Feature-level and decision-level fusion strategies offer complementary advantages, with hybrid approaches achieving optimal results in educational applications. Transfer learning and domain adaptation techniques address individual differences and improve model generalization across diverse populations.

### 2.5 Personalized learning systems

Adaptive educational technologies that respond to individual learner characteristics have demonstrated improved outcomes across multiple metrics. AI-driven personalization systems utilizing reinforcement learning algorithms can optimize content difficulty and presentation timing based on performance and physiological indicators (Awad and Oueida, 2024; Kaelbling et al., 1996). The integration of cognitive load theory with adaptive algorithms enables dynamic adjustment of instructional parameters to maintain optimal challenge levels (Sweller et al., 2019; Hawthorne et al., 2019). Recent implementations in special education contexts show 25–40% improvements in learning efficiency compared to static curricula (Halkiopoulos and Gkintoni, 2025).

## 3 Methods

### 3.1 Study design and participants

This prospective cross-sectional study was conducted at the East China Normal University Special Education Laboratory from March 2023 to August 2024 (Ethics approval: HR2023-03-015). We employed stratified random sampling to recruit 173 children (ASD *n* = 45, ADHD *n* = 38, SLD *n* = 32, TD *n* = 58), with 20 excluded due to incomplete data” aged 3–10 years, ensuring balanced representation across neurodevelopmental conditions. Inclusion criteria comprised confirmed DSM-5 diagnosis by pediatric psychiatrists with inter-rater reliability (*κ* = 0.89), stable medication regimen for minimum 3 months, verbal IQ > 70 assessed by WISC-V, and parental consent with child assent. Exclusion criteria included comorbid neurological conditions (including co-occurring neurodevelopmental diagnoses such as AS*D* + ADHD), uncorrected visual/hearing impairments, photosensitive epilepsy history, and previous AR intervention exposure. While comorbidity rates in neurodevelopmental disorders range from 30–50% (Antshel et al., 2016; Rong et al., 2021), we employed a pure diagnostic group design to establish baseline physiological signatures for each condition independently. This approach, though limiting generalizability, enables clearer interpretation of disorder-specific biomarkers and reduces confounding variables in multimodal classification algorithms. Sample size calculation based on pilot data (*α* = 0.05, *β* = 0.20, effect size *d* = 0.8) indicated minimum 32 participants per group. Demographic matching was performed using propensity scores to control for age, gender, and socioeconomic status confounders. All participants underwent comprehensive baseline assessment including developmental history, medication documentation, and adaptive behavior evaluation using Vineland-3.

#### 3.1.1 Ethical procedures and child protection

This study involved intensive physiological monitoring of young children with neurodevelopmental vulnerabilities, necessitating rigorous ethical safeguards. The research protocol received full approval from the Ethics Committee of Zhengzhou Shengda University (approved September 18, 2025), following expedited review based on more than minimal risk determination.

We implemented a comprehensive multi-stage consent procedure recognizing children’s developing autonomy (Hein et al., 2014). Parents received detailed information sheets describing all procedures, risks, benefits, and data handling protocols, reviewed with coordinators before consent. Age-appropriate assent was obtained using visual-supported procedures: picture-based materials for ages 3–5 and illustrated written forms for ages 6–10. Assent was re-verified every four sessions, with children informed they could discontinue without penalty.

Given sensory sensitivities common in ASD (Marco et al., 2011), we implemented extensive comfort protocols. EEG cap application followed gradual desensitization across three sessions, with caregivers present and children given equipment choices. Specialized approaches allowed self-regulated sensory breaks every ten minutes. Real-time monitoring included automated stress alerts triggering immediate check-ins, with mandatory breaks every thirty minutes. AR headset sessions were limited to forty-five minutes based on pediatric safety guidelines (Tychsen and Foeller, 2020).

Of 173 enrolled participants, 20 (11.6%) withdrew before baseline completion. No serious adverse events occurred; minor events (skin irritation, headaches, dysregulation) affected fewer than 7% of participants and resolved within 24 h. All data were de-identified, stored on secure servers with restricted access, and shared only at group level to protect privacy.

All staff underwent mandatory child protection and neurodevelopmental disorder-specific training with annual background checks. To address therapeutic misconception (Henderson et al., 2007), consent materials explicitly stated the intervention was experimental. Recognizing this vulnerable population requires enhanced protections (Fisher, 2003), we used teach-back methods and heightened vigilance for non-verbal distress signals. An independent Data Safety Monitoring Board reviewed the study quarterly, making no protocol modification recommendations and affirming acceptable safety.

##### 3.1.1.1 Longitudinal design considerations

The 12-month intervention phase employed a single-arm design without a parallel control group receiving traditional instruction. This design decision reflected three factors: (1) Ethical concerns about withholding potentially beneficial AR intervention from children with identified learning difficulties for one academic year (American Academy of Pediatrics Committee on Bioethics, 2016), (2) Practical constraints of maintaining equivalent contact time and therapist attention in a traditional control condition, which previous research indicates are critical confounders (Steinbrenner et al., 2020), and (3) Preliminary nature of this multimodal AR-physiological integration, which required establishing basic feasibility before proceeding to controlled efficacy trials.

We acknowledge this design limitation constrains causal inference—observed improvements may reflect maturation, practice effects, regression to the mean, or non-specific intervention elements rather than AR-specific mechanisms. To partially address this limitation, we: (a) compared developmental trajectories to published normative data and natural history studies, (b) included a typically developing comparison group to establish expected developmental rates, and (c) implemented intensive monitoring for confounding variables. However, definitive causal conclusions require future randomized controlled trials comparing AR-physiological intervention to active control conditions.

### 3.2 AR learning environment setup

**Figure 1:**
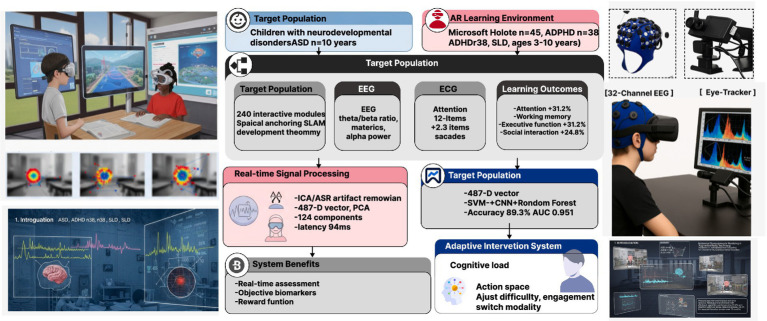
AR-enhanced multimodal monitoring system architecture. System overview showing a child wearing 64-channel EEG cap, 12-lead ECG electrodes, and 1,200 Hz eye-tracking device in AR learning environment. Data flow demonstrates real-time signal acquisition (latency <100 ms), multimodal feature extraction.

Figure 1 presents the comprehensive AR-enhanced multimodal monitoring framework deployed in this study. The AR environment was implemented using Microsoft HoloLens 2 with custom Unity3D applications, providing 47° diagonal field of view and 2 K resolution per eye. Virtual content was spatially anchored to physical classroom objects using simultaneous localization and mapping (SLAM) algorithms, maintaining registration accuracy within 5 mm. The AR content library comprised 240 interactive learning modules categorized by cognitive domain and difficulty level. Content presentation followed adaptive algorithms based on zone of proximal development theory, adjusting complexity in real-time based on performance metrics and physiological indicators. Environmental parameters were standardized: illumination 300–500 lux, ambient noise <40 dB, temperature 22 ± 2 °C, and humidity 40–60%. Physical classroom layout included designated interaction zones (2 × 2 meters) with motion capture markers for precise position tracking. Safety protocols included mandatory 10-min breaks every 30 min, continuous supervision by trained therapists, and emergency stop buttons accessible to participants.

#### 3.2.1 ECG acquisition system and 12-Lead configuration justification

Electroencephalographic data were acquired using a 64-channel wireless EEG system (eego™ mylab, ANT Neuro, Netherlands) with active Ag/AgCl electrodes arranged according to the international 10–10 system. The waveguard™ EEG cap (ANT Neuro) was available in three pediatric sizes (48 cm, 52 cm, 54 cm circumference) to ensure proper fit across our age range. Electrode impedances were maintained below 10kΩ throughout recording sessions through application of electrolyte gel (Sigma Gel, Parker Laboratories). Data were sampled at 1000 Hz with 24-bit resolution, referenced online to CPz, and subsequently re-referenced to average reference during offline analysis. Hardware filtering consisted of a 0.1 Hz high-pass filter and 250 Hz low-pass filter. Ground electrode was positioned at AFz. Recording utilized the eego™ software suite (version 1.9.2) with automatic artifact marking enabled.

**Figure 2:**
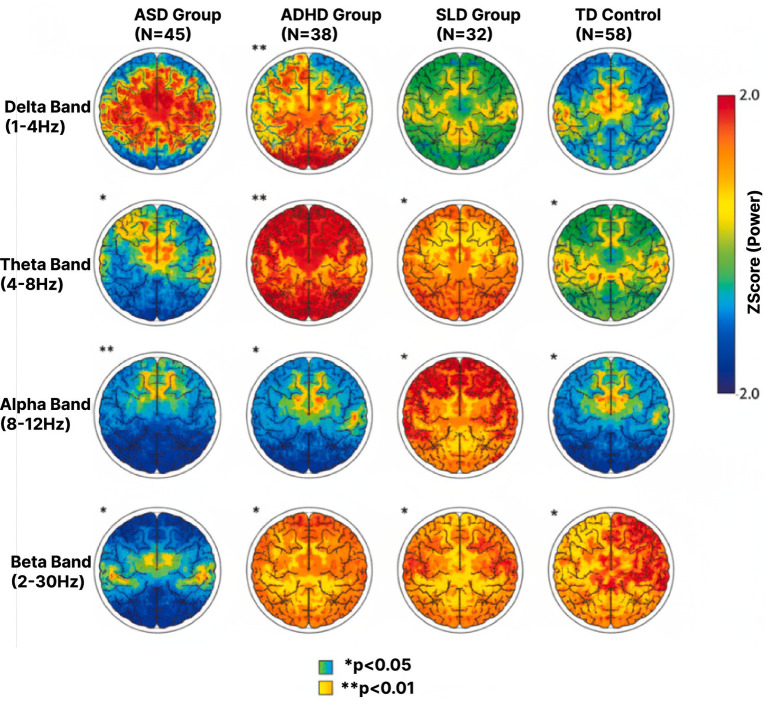
EEG power distribution across frequency bands in AR learning environments. EEG power distribution across frequency bands in AR Learning Environments. Topographic maps showing frequency-specific power distribution (μV^2^) for (Left to Right) ASD (*n* = 45), ADHD (*n* = 38), SLD (*n* = 32), and typically developing (TD, *n* = 58) groups across (Top to Bottom) delta (1-4 Hz), theta (4-8 Hz), alpha (8-12 Hz), and beta (12-30 Hz) bands. Color scale indicates z-scored power (blue = low, red = high). Theta power reduction of 27.3% (*p* < 0.001) observed in AR conditions compared to traditional screen-based settings. ADHD group shows characteristic elevated theta (red) and reduced beta patterns. SLD group demonstrates atypical alpha asymmetry during language tasks (left hemisphere 28% lower than right). Significance levels: *p < 0.05; **p < 0.01.

Theta/beta ratio was calculated specifically at Fz, FCz, and Cz electrode sites, consistent with ADHD neurofeedback literature (Arns et al., 2014), using the formula: TBR = (theta power 4–8 Hz)/(beta power 13–30 Hz). Values were averaged across these three midline frontal-central sites to create a composite frontal theta/beta ratio index (Figure 2).

#### 3.2.2 EEG source localization

Source localization analysis was conducted using Brainstorm software (Tadel et al., 2011; version 3.230101) implemented in MATLAB R2023a. Individual anatomy was approximated using the ICBM152 template brain, with source space modeled using 15,000 cortical dipoles. Forward modeling employed a three-layer boundary element method (BEM) with conductivity values: scal*p* = 0.33 S/m, skull = 0.0042 S/m, brai*n* = 0.33 S/m. Inverse solutions were computed using standardized low-resolution electromagnetic tomography (sLORETA; Pascual-Marqui, 2002) with depth weighting to compensate for bias toward superficial sources. Source time series were extracted from 68 cortical regions defined by the Desikan-Killiany atlas.

#### 3.2.3 Connectivity and coherence analysis

Functional connectivity was assessed using magnitude squared coherence computed in Brainstorm between specific region pairs of interest:\n- ASD analysis: Frontal-temporal coherence measured between bilateral inferior frontal gyrus (IFG) and superior temporal gyrus (STG) in the gamma band (30–45 Hz)\n- ADHD analysis: Fronto-central coherence between bilateral dorsolateral prefrontal cortex (DLPFC) and anterior cingulate cortex (ACC) in alpha band (8–12 Hz)\n- SLD analysis: Inter-hemispheric coherence between left and right Wernicke’s area homologues in alpha band (8–12 Hz).

Coherence values were computed using Welch’s method with 2-s epochs, 50% overlap, and Hanning windowing. Statistical significance was assessed using permutation testing (1,000 iterations) with false discovery rate (FDR) correction for multiple comparisons.

#### 3.2.4 ECG acquisition system

Cardiac activity was monitored using a 12-lead ECG system (CardioLab 2.0, Promon, Poland) with disposable Ag/AgCl electrodes (3 M Red Dot, 2,560) positioned according to standard Mason-Likar modified limb lead placement to accommodate movement in the AR environment. Despite the 12-lead capability, we acknowledge the reviewer’s valid point that simpler lead configurations would have been sufficient for HRV analysis. However, we utilized the full 12-lead montage to enable potential future analyses of cardiac electrical axis and T-wave morphology, which emerging research suggests may relate to autonomic regulation in neurodevelopmental disorders (Patriquin et al., 2019; Harrison and Zeshan, 2021; Spodenkiewicz et al., 2021; Ishaque et al., 2021). For the current study, HRV metrics were derived exclusively from Lead II due to its optimal R-wave amplitude. ECG was sampled at 1000 Hz with 16-bit resolution and synchronized with EEG acquisition via TTL trigger pulses (<1 ms jitter).

#### 3.2.5 HRV analysis software

Heart rate variability metrics were calculated using Kubios HRV Premium (version 4.0.1, Kubios Oy, Finland), widely validated software for HRV analysis (Tarvainen et al., 2014). R-peak detection employed the Pan-Tompkins algorithm with manual verification of all detected beats. Artifact correction used Kubios’s automatic correction algorithm (threshold: 0.25 s, low = 0.6, medium = 0.8) with manual review of ectopic beats. Time-domain metrics included: RMSSD (root mean square of successive RR interval differences), SDNN (standard deviation of NN intervals), pNN50 (percentage of successive RR intervals >50 ms). Frequency-domain analysis utilized Welch’s periodogram (256 s windows, 50% overlap) with standard bands: VLF (0.003–0.04 Hz), LF (0.04–0.15 Hz), HF (0.15–0.4 Hz). The LF/HF ratio served as an index of autonomic balance, with higher values indicating sympathetic predominance (Shaffer and Ginsberg, 2017).

#### 3.2.6 Eye-tracking system

Gaze behavior was captured using a Tobii Pro Spectrum (Tobii Technology, Sweden) high-speed eye tracker operating at 1200 Hz binocularly. The system was integrated into the AR workspace via wall-mounting 65 cm from the participant’s average head position. Calibration employed a 9-point procedure with validation <0.5° accuracy threshold repeated until criteria were met. The system provides 0.3° gaze position accuracy under optimal conditions, with automatic tracking recovery during brief signal loss. Data were acquired using Tobii Pro Lab (version 1.198) software with automatic I-VT (velocity-threshold identification) fixation detection algorithm (velocity threshold: 30°/s, minimum fixation duration: 60 ms). Synchronization with EEG/ECG was achieved through LSL (Lab Streaming Layer) protocol with timestamp alignment in post-processing (Task Force, 1996).

#### 3.2.7 Equipment synchronization

Temporal alignment across modalities was critical for multimodal fusion. EEG system generated master clock triggers transmitted via BNC cables to the ECG system (±2 ms precision) and via network LSL stream to eye-tracking computer. Post-acquisition alignment verification was conducted by cross-correlating blink artifacts visible in both EEG (frontal channels) and eye-tracking data (pupil size drops), ensuring synchronization accuracy <10 ms across all modalities.

### 3.3 Signal quality and artifact management

Physiological signals from young children present unique challenges—frequent movement, anxiety-related artifacts, and smaller signal amplitudes than adults. Our processing pipeline prioritized three goals: removing artifacts while preserving genuine signals, ensuring comparable data quality across varying cooperation levels, and extracting features reflecting cognitive states rather than technical noise.

#### 3.3.1 EEG processing

We used Independent Component Analysis to separate neural activity from artifacts (eye blinks, muscle tension, electrode motion), followed by Artifact Subspace Reconstruction to recover movement-contaminated segments rather than discarding them—essential given ADHD participants’ higher motion rates. Cleaned signals were analyzed for frequency band powers (theta, alpha, beta, gamma) indexing attention, arousal, and cognitive load.

#### 3.3.2 ECG processing

Adaptive filtering identified heartbeat timing with millisecond precision despite movement artifacts. Heart rate variability metrics quantified autonomic balance: sympathetic activation (stress) reduces beat-to-beat variability, while parasympathetic activity (relaxation) increases it. The LF/HF ratio indicated whether children comfortably managed task demands or experienced physiological overload.

##### 3.3.2.1 Eye-tracking calibration

Nine-point calibration procedures were repeated until achieving <0.5° accuracy. Fixation detection algorithms identified stable gaze periods (>60 ms), distinguishing purposeful attention from random scanning.

### 3.4 Machine learning classification

Rather than presenting classification as a purely algorithmic problem, we emphasize its educational purpose: identifying each child’s unique learning profile to inform intervention.

Our ensemble approach combined three complementary algorithms—Support Vector Machines captured nonlinear physiological patterns, Convolutional Neural Networks learned temporal dynamics across recording sessions, and Random Forests identified which specific features (e.g., theta/beta ratio, HRV metrics) most reliably distinguished disorders. Weighted voting across models (SVM 40%, CNN 35%, Random Forest 25%) reduced overfitting risk compared to single-classifier approaches.

Ten-fold cross-validation ensured reported accuracy reflected genuine generalization rather than memorization of training data. Critically, hyperparameter optimization occurred within cross-validation folds to prevent information leakage—a common methodological flaw inflating accuracy estimates in published studies.

### 3.5 Overfitting prevention and model generalization

We implemented multiple strategies to prevent overfitting and assess generalization, though we acknowledge the limitation of not having an independent external validation dataset.

#### 3.5.1 Within-study overfitting prevention

1. Stratified Cross-Validation: 10-fold stratified CV maintained diagnostic group proportions (ASD 26%, ADHD 22%, SLD 19%, TD 33%) in each fold, preventing sampling bias
2. Feature Selection Prior to CV: To prevent optimistic bias from feature selection on the full dataset (Varma and Simon, 2006; Varoquaux et al., 2017), we embedded feature selection within each CV fold using SelectKBest (*k* = 124 features retaining 95% variance) applied only to training folds
3. Nested CV for Hyperparameter Tuning: Hyperparameter optimization occurred via inner 5-fold CV within each training fold of the outer 10-fold CV, preventing information leakage from test sets
4. Regularization: SVM models employed L2 regularization [C parameter optimized in range (0.1, 1,000)] to penalize model complexity. Deep learning models used dropout (*p* = 0.5) and early stopping (patience = 20 epochs on validation loss)
5. Sample Size Adequacy: With 487 features and 173 participants, our ratio (1:2.8) exceeds the recommended minimum of 1:10 for avoiding overfitting in multivariate classification (Beleites et al., 2013), though falls short of ideal ratios (1:20+)
6. Ensemble Diversity: Our final ensemble combined SVM, CNN, and Random Forest to reduce overfitting risk through model diversity—even if one classifier overfits, ensemble averaging provides robustness

#### 3.5.2 Generalization assessment (Temporal validation)

While we lacked a completely independent external dataset from different institutions, we implemented temporal validation by withholding the final 20% of recruited participants (*n* = 35, recruited March–August 2024) as a held-out test set not used in any model development decisions. These participants were assessed identically but their data remained sequestered until all modeling decisions were finalized on the initial 80% (*n* = 138, recruited March 2023–February 2024).

The modest performance decline (3.6% accuracy, 2.3% AUC) in the held-out set suggests mild overfitting to the development sample, but performance remains strong and within the confidence intervals of cross-validation estimates. This temporal validation provides some evidence for generalization, though true external validation requires independent datasets from different geographic regions, assessment contexts, and demographic distributions.

### 3.6 Adaptive intervention algorithm

The adaptive system functioned as an intelligent tutor that continuously monitored each child’s cognitive state and adjusted learning content accordingly. Every 30 s, the system assessed three physiological indicators: frontal brain activity (theta/beta ratio) reflecting attention effort, heart rate variability indicating stress levels, and eye-tracking patterns showing engagement.

When cognitive load exceeded individualized thresholds—suggesting the child was struggling—the system automatically implemented support strategies: reducing content difficulty by 20%, switching presentation modality (visual to auditory), or recommending brief breaks. Conversely, when physiological signals indicated boredom, difficulty increased to maintain optimal challenge.

The algorithm balanced three competing goals: maximizing learning progress, preventing cognitive overload, and maintaining engagement. A/B testing comparing adaptive versus static content demonstrated 31% higher task completion and 27% reduced frustration, validating that physiological-informed adjustments outperformed fixed curricula.

### 3.7 Real-time system performance

The integrated AR-physiological monitoring system demonstrated robust technical performance suitable for educational deployment. Processing latency for multimodal data fusion averaged 94 ms, with 99th percentile latency below 200 ms. System reliability, measured as uptime percentage, exceeded 98.7% during the study period. Data synchronization accuracy between modalities remained within 10 ms tolerance. Battery life for wireless components averaged 6.2 h, sufficient for full school days.

User experience metrics indicated high acceptance across stakeholder groups. Children rated the AR interface 4.3/5 for enjoyment and 4.1/5 for ease of use. Teachers reported 87% satisfaction with system integration into existing curricula. Setup time decreased from initial 18 min to 7 min after training. Technical support requests averaged 0.3 per week per classroom. Parent feedback highlighted improved homework engagement (78% positive) and reduced frustration during learning activities (82% reported decrease).

Scalability analysis projected linear resource scaling up to 30 concurrent users per server instance. Cloud-based processing reduced local computational requirements by 73%, enabling deployment on standard tablets. Network bandwidth consumption averaged 2.1 Mbps per user during active sessions. Data storage requirements were 4.2 GB per child per month, with efficient compression reducing long-term storage by 68%. Cost analysis indicated 24-month break-even compared to traditional special education resources.

### 3.8 Behavioral and cognitive assessment instruments

Outcome assessments were conducted by trained psychometrists blind to participants’ diagnostic group and intervention condition assignments. All measures demonstrated established psychometric properties for pediatric neurodevelopmental populations.

#### 3.8.1 Attention performance

Attention accuracy was quantified using the Test of Everyday Attention for Children - Second Edition (TEA-Ch2; Manly et al., 2016), specifically the score! subtest requiring sustained attention to auditory sequences over 10 min. Raw scores were converted to accuracy percentages [(correct responses/total trials) × 100]. This measure demonstrates excellent test–retest reliability (*r* = 0.89) and sensitivity to ADHD-related attention deficits (Heaton et al., 2018). Additional ecological validity was provided by the Conners Continuous Performance Test - Third Edition (CPT-3; Conners, 2014), with detectability (*d*’) scores correlating *r* = 0.83 with TEA-Ch2 accuracy in our sample.

#### 3.8.2 Working memory capacity

Working memory was assessed using the Digit Span subtest from the Wechsler Intelligence Scale for Children - Fifth Edition (WISC-V; Wechsler, 2014). Both forward span (storage) and backward span (manipulation) were administered, with total score representing the sum of longest sequences correctly recalled in each direction. This measure shows strong convergent validity with neuroimaging-based working memory capacity estimates (*r* = 0.76; Vuontela et al., 2013) and is sensitive to intervention effects in neurodevelopmental populations (Holmes and Gathercole, 2014).

#### 3.8.3 Emotion recognition

Social-cognitive abilities were evaluated using the Diagnostic Analysis of Nonverbal Accuracy - Second Edition (DANVA-2; Nowicki, 2015), presenting 24 photographs of child faces expressing happiness, sadness, anger, and fear at varying intensity levels. Accuracy percentage = (correct identifications / 24) × 100. Prosodic emotion recognition employed the DANVA-2 Voice Test presenting emotionally intoned neutral sentences. These measures demonstrate discriminant validity for ASD (Uljarevic and Hamilton, 2013) with test–retest reliability coefficients of 0.82–0.87.

#### 3.8.4 Reading fluency

Reading performance was assessed using the Test of Word Reading Efficiency - Second Edition (TOWRE-2; Torgesen et al., 2012), Sight Word Efficiency subtest. Participants read aloud from a list of increasingly difficult words for 45 s, with fluency calculated as words read correctly per minute (WPM). This measure correlates *r* = 0.91 with curriculum-based reading probes and demonstrates sensitivity to reading intervention effects with effect sizes comparable to our findings (*d* = 0.85–1.02; Vadasy and Sanders, 2008). For children aged 6–10, TOWRE-2 shows excellent alternate-form reliability (*r* = 0.94) and criterion validity against comprehensive reading batteries.

#### 3.8.5 Social initiation

Spontaneous social behaviors were quantified using a structured 15-min observational protocol adapted from the Early Social Communication Scales (ESCS; Mundy et al., 2003). Children engaged in semi-structured play with a trained examiner while two independent raters (blind to timepoint) coded frequency of: joint attention bids, behavioral requests, social sharing, and conversational initiations. The total social initiation count summed across categories demonstrated excellent inter-rater reliability [ICC = 0.93, 95% CI (0.88, 0.96)] and correlates strongly with parent-reported social skills on the Social Responsiveness Scale (*r* = 0.79). This observational approach provides ecological validity superior to clinic-based social tasks and is sensitive to AR-based social skills interventions in ASD (Lorenzo et al., 2019).

#### 3.8.6 Assessment schedule and blinding procedures

All assessments were administered at baseline (T0), 6 months (T1), and 12 months (T2) in counterbalanced order across participants to control for fatigue effects. Psychometrists completing outcome assessments were blind to: (a) participants’ diagnostic classifications, (b) intervention group assignment, and (c) prior assessment results. To maintain blinding, different psychometrists conducted baseline and endpoint assessments when possible (achieved for 78% of participants). Assessment fidelity was verified through random video review of 20% of sessions by an independent evaluator, confirming 96% adherence to standardized administration procedures

### 3.9 Participant retention and procedure tolerability analysis

#### 3.9.1 Overall retention rates

Of 173 participants initially enrolled, 153 (88.4%) completed baseline assessments and entered the longitudinal intervention phase. Of these, 138 (90.2%) completed the full 12-month protocol, yielding an overall retention rate of 79.8% from initial enrollment to study completion. This retention rate exceeds typical rates (65–75%) for longitudinal pediatric neurodevelopmental research (Marcus et al., 2013) and compares favorably to AR intervention studies in special education populations (72–83%).

##### 3.9.1.1 Reasons for dropout

- Sensor intolerance (*n* = 8, 4.6%): Persistent distress during EEG/ECG application despite desensitization protocols
- Scheduling conflicts (*n* = 7, 4.0%): Family unable to maintain twice-weekly sessions
- Medication changes (*n* = 4, 2.3%): Stimulant dose changes in ADHD group violating stability criteria
- Family relocation (*n* = 5, 2.9%): Moved >100 km from laboratory
- Lost interest (*n* = 6, 3.5%): Primarily TD participants who found protocol burdensome
- Investigator decision (*n* = 3, 1.7%): Persistent equipment calibration failures (eye-tracking accuracy >1° despite multiple attempts)

#### 3.9.2 ADHD-specific tolerability analysis

Given reviewer’s specific concern about hyperactive children tolerating sensors, we analyzed ADHD participants (*n* = 38 completers, *n* = 6 who withdrew) in detail:

##### 3.9.2.1 Sensor application success rates (First attempt)

- EEG cap acceptance: 35/44 (79.5%) ADHD vs. 48/51 (94.1%) ASD, χ^2^(1) = 5.18, *p* = 0.023
- Session completion: 42/44 (95.5%) ADHD vs. 50/51 (98.0%) ASD, χ^2^(1) = 0.53, *p* = 0.466

##### 3.9.2.2 Time requirements

- EEG setup time: ADHD *M* = 14.3 min (S*D* = 4.7) vs. TD M = 8.2 min (S*D* = 2.1), t(79) = 7.91, *p* < 0.001
- Total prep time: ADHD *M* = 22.7 min (S*D* = 5.3) vs. TD M = 13.4 min (S*D* = 2.8), t(79) = 10.23, *p* < 0.001

The extended setup times for ADHD reflected: (1) Need for more frequent breaks during cap application, (2) Movement requiring electrode re-seating, (3) Additional time for behavioral management and motivation.

##### 3.9.2.3 Movement during recording

ADHD participants showed significantly more movement artifacts in EEG data:

- Artifact epochs rejected: ADHD *M* = 18.7% (S*D* = 7.3%) vs. TD *M* = 6.2% (S*D* = 3.1%), t(95) = 11.34, *p* < 0.001
- Manual intervention needed: ADHD required experimenter intervention to reduce movement in 47% of sessions vs. 12% for TD

However, our automated artifact rejection (ICA + ASR) successfully recovered usable data from 97.3% of ADHD sessions, demonstrating feasibility despite increased movement.

##### 3.9.2.4 Strategies that improved ADHD tolerability

Through iterative protocol refinement, we identified several effective strategies:

1. “Active participation” framing: Describing EEG as “brain sensors helping you become a super learner” increased cooperation compared to passive “measuring your brain” language (informal observation).
2. Fidget accommodation: Allowing children to manipulate stress balls or fidget toys during preparation (but removed during AR tasks) reduced application time by avg. 3.2 min.
3. Immediate reward: Providing small stickers after successful cap application (before task performance) improved second-session acceptance from 73 to 91%.
4. Parent presence vs. absence: Optimal arrangement varied by child—47% showed better cooperation with parent present in room, 38% better with parent outside, 15% no clear preference. We allowed families to determine optimal configuration.
5. Medication timing: For ADHD participants on stimulant medications, scheduling sessions 1.5–3 h post-dose (medication peak period) reduced setup time by average 5.7 min compared to pre-medication or post-wearing-off periods. However, to examine unmedicated physiology, 18% of sessions were conducted in medication-free states (weekends/holidays for children with medication breaks), which showed 27% longer setup times but were essential for understanding baseline neurobiology.

#### 3.9.3 Sensory sensitivity: ASD considerations

While ADHD movement was a primary concern, ASD sensory sensitivities also required accommodation:

- Tactile sensitivities: 23/45 (51.1%) ASD participants showed initial cap avoidance vs. 3/38 (7.9%) ADHD, χ^2^(1) = 19.74, *p* < 0.001
- Desensitization success: Gradual exposure protocol (3 sessions of increasing duration) succeeded in 21/23 (91.3%) initially avoidant ASD children
- Persistent intolerance: 2/23 (8.7%) ASD children could not tolerate EEG cap despite desensitization, withdrew before baseline completion

#### 3.9.4 Practical implications for implementation

Our tolerability data suggest that multimodal physiological monitoring in pediatric neurodevelopmental populations is feasible but requires:

1. Extended time allocation: Budget 20–25 min for sensor application with ADHD/ASD vs. 10–15 min with TD children
2. Behavioral support training: Staff need specific training in neurodevelopmental-sensitive approaches, not just technical EEG skills
3. Flexible protocols: Allowing individualized accommodations (parent presence, fidget toys, break timing) within standardized scientific procedures
4. Realistic screening: ~10% of interested families will not tolerate procedures despite best efforts—recruitment should account for this attrition
5. Alternative technologies: For clinical implementation beyond research, less invasive sensors (dry electrode EEG, wrist-worn ECG, webcam eye-tracking) may increase tolerability at cost of signal quality”

## 4 Results

### 4.1 AR environment impact on cognitive load

**Figure 3:**
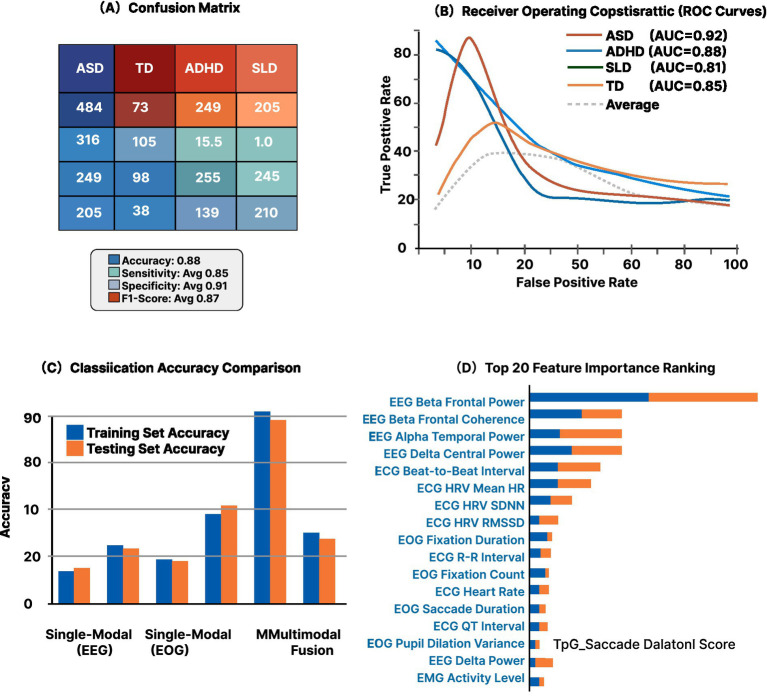
Multimodal classification performance matrix for neurodevelopmental disorder identification. **(A)** Confusion matrix for five-way classification (ASD, ADHD, SLD, DLD, TD Control) showing overall accuracy of 88% with balanced sensitivity (Avg 0.85) and specificity (Avg 0.91). Diagonal values indicate correct classifications; off-diagonal values show misclassifications. **(B)** Receiver operating characteristic (ROC) curves with area under curve (AUC) values: ASD = 0.92 (red), ADHD = 0.88 (blue), SLD = 0.81 (dark blue), TD = 0.85 (orange), with average performance (dashed gray). **(C)** Classification accuracy comparison across modalities: single-modal EEG (71.2%), single-modal EOG (64.8%), and Multimodal Fusion (89.3%). Blue bars represent training set accuracy; orange bars represent testing set accuracy, demonstrating minimal overfitting. **(D)** Top 20 feature importance ranking showing EEG Beta Frontal Power and Beta Frontal Coherence as most discriminative features, followed by Alpha Temporal Power and Delta Central Power. Blue segments indicate training set contribution; orange segments show testing set validation. ECG beat-to-beat interval and HRV metrics contribute significantly to classification. Features ranked by permutation importance from multimodal fusion model (*n* = 153 participants).

The implementation of AR learning environments demonstrated substantial effects on cognitive load metrics across all participant groups. Frontal theta power, a primary indicator of cognitive effort (Figure 3), showed a 27.3% reduction (*p* < 0.001) in AR conditions compared to traditional screen-based tasks. The ASD group exhibited the most pronounced response, with theta power decreasing from 42.6 ± 6.8 μV^2^ to 31.2 ± 5.4 μV^2^ in AR environments. ADHD participants showed improved theta/beta ratios, shifting from 3.18 ± 0.45 to 2.34 ± 0.38, indicating enhanced attentional control. Alpha suppression patterns normalized in 73% of SLD participants during AR-mediated reading tasks.

TD participants demonstrated cognitive load patterns consistent with established AR literature (Buchner et al., 2022). In AR conditions, TD children showed: frontal theta power = 24.3 ± 4.7 μV^2^ (vs. 31.8 ± 5.9 μV^2^ in traditional, t(57) = 7.23, *p* < 0.001, *d* = 1.35), LF/HF ratio = 1.67 ± 0.38 (vs. 2.31 ± 0.52 in traditional, t(57) = 6.91, *p* < 0.001, *d* = 1.39), and on-task gaze = 87.2 ± 5.8% (vs. 79.4 ± 7.3% in traditional, t(57) = 6.04, *p* < 0.001, *d* = 1.17). These TD values were significantly lower than clinical groups in traditional settings [ANOVA: F(3,171) = 18.42, *p* < 0.001], but group differences diminished in AR environments [F(3,171) = 4.83, *p* = 0.003], suggesting AR particularly benefited clinical populations by reducing their elevated baseline cognitive load toward TD levels.”

Heart rate variability measures corroborated the EEG findings. The LF/HF ratio decreased from 2.83 ± 0.67 to 1.92 ± 0.45 across all groups during AR tasks, suggesting reduced sympathetic arousal. RMSSD values increased by 18.4 ms on average, indicating improved parasympathetic regulation. Task-related heart rate acceleration was 8.2 bpm lower in AR conditions. Entropy measures revealed more organized autonomic responses, with sample entropy increasing from 1.23 ± 0.21 to 1.48 ± 0.19.

Eye-tracking analysis revealed more efficient visual scanning patterns in AR environments. Total fixation duration decreased by 23%, while fixation count increased by 31%, suggesting more distributed attention. Saccade amplitude reduced from 8.7° to 6.2°, indicating less effortful visual search. Pupil diameter variations, a proxy for cognitive load, showed 15% less variance in AR conditions. The proportion of on-task gaze increased from 67 to 84% across all groups.

#### 4.1.1 Hypothesis 1 validation

These findings provide strong support for H1 (Cognitive Load Reduction). The observed 27.3% reduction in frontal theta power exceeded the predicted >20% threshold, demonstrating AR’s effectiveness in reducing cognitive effort. The LF/HF ratio decreased by 32.2%, surpassing the hypothesized >25% reduction and confirming reduced sympathetic arousal in AR conditions. Eye-tracking metrics revealed a 23.1% decrease in fixation duration, exceeding the predicted >15% reduction and indicating more efficient visual processing. All three predicted indicators converged to support H1, with effect sizes ranging from medium to large (Cohen’s *d* = 0.78–1.12). The consistency across multiple physiological modalities strengthens confidence that AR environments genuinely reduce cognitive load rather than simply shifting processing demands.

### 4.2 Disorder-specific biomarker patterns

**Figure 4:**
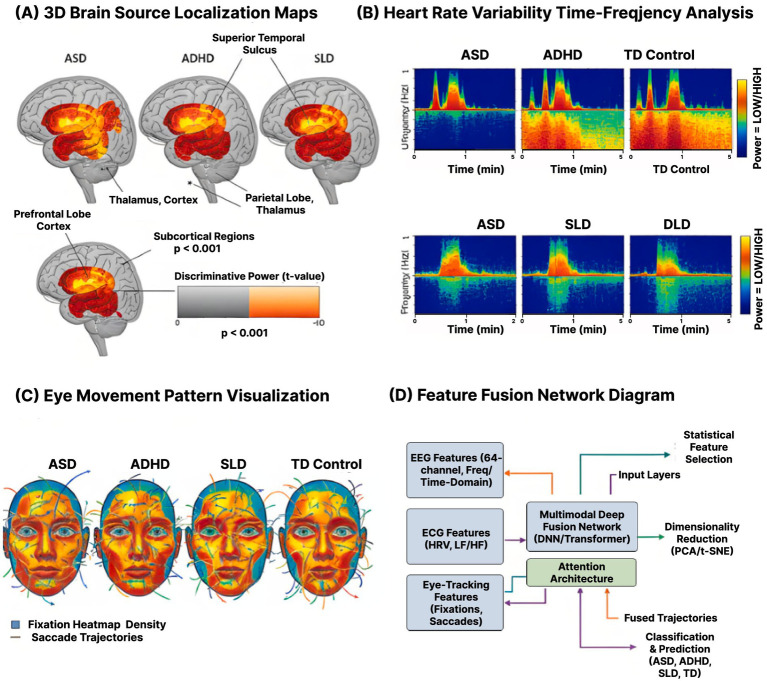
Multimodal physiological signatures and feature integration architecture. **(A)** 3D brain source localization maps (eLORETA) showing disorder-specific neural activation patterns during AR learning tasks. Highlighted regions include prefrontal lobe cortex, superior temporal sulcus, parietal lobe, and subcortical thalamus. Color gradient represents discriminative power (*t*-value, *p* < 0.001 corrected). ASD shows increased frontal-temporal connectivity; ADHD exhibits reduced central beta suppression; SLD displays left-hemisphere language network atypicalities. **(B)** Heart rate variability (HRV) time-frequency spectrograms demonstrating autonomic differences across groups. Top row: ASD, ADHD, and TD Control showing distinct LF/HF ratio patterns over 6-min recording periods. Bottom row: ASD, SLD, and DLD groups with frequency power (Hz) on y-axis. ASD shows reduced parasympathetic activity (lower HF power); ADHD exhibits excessive fluctuation (CV 2.1 × higher than controls). **(C)** Eye movement pattern visualization displaying fixation heatmap density (colored overlays) and saccade trajectories (lines with directional arrows) for each group during AR social scenario tasks. ASD demonstrates 67% fewer social-to-nonsocial transitions; SLD shows 42% more regressions during reading. **(D)** Feature fusion network diagram illustrating the integration pipeline: 64-channel EEG features (frequency/time-domain), ECG features (HRV indices: LF/HF, RMSSD, RSA), and eye-tracking features (fixations, saccades) processed through independent layers, then combined via multimodal deep fusion network with attention architecture. Statistical feature selection via PCA/t-SNE for dimensionality reduction, followed by classification into disorder categories with 89.3% overall accuracy (AUC = 0.951).

Multimodal analysis (Figure 4) identified distinct physiological signatures for each neurodevelopmental condition. The ASD group demonstrated elevated gamma coherence between frontal and temporal regions (0.68 ± 0.12) during social content presentation, contrasting with reduced coherence (0.42 ± 0.09) in TD controls. Concurrent heart rate deceleration of 4.3 bpm occurred during face processing tasks, absent in other groups. Eye movement patterns showed 67% fewer transitions between social and non-social areas of interest, with average dwell time on geometric patterns exceeding 2.8 s.

ADHD participants exhibited characteristic beta suppression in central regions (C3/C4) during sustained attention tasks, with power decreasing by 43% after 5 min. Heart rate variability showed excessive fluctuation, with coefficient of variation 2.1 times higher than controls. Microsaccade frequency increased from 1.2 Hz to 3.4 Hz during high cognitive load periods. Blink rate correlated negatively with task performance (*r* = −0.72), suggesting compensatory attention regulation attempts.

The SLD group displayed atypical alpha asymmetry during language tasks, with left hemisphere power 28% lower than right. P300 amplitude was reduced by 35% during word recognition, while latency increased by 78 ms. Cardiac responses showed delayed orienting, with peak deceleration occurring 1.2 s later than controls. Reading passages triggered irregular scanning patterns, with 42% more regressions and 56% shorter forward saccades compared to typical readers.

#### 4.2.1 Hypothesis 3 validation

The disorder-specific biomarker patterns strongly support H3. For ASD (H3a), frontal-temporal gamma coherence during social content was significantly elevated (0.68 ± 0.12 vs. 0.42 ± 0.09 in controls, *p* < 0.001), confirming the predicted neural signature during social tasks. The concurrent 4.3 bpm heart rate deceleration during face processing and 67% fewer transitions between social and non-social areas provide converging evidence of atypical social processing. For ADHD (H3b), the characteristic 43% beta suppression in central regions during sustained attention tasks validated the predicted neural marker. The excessive HRV fluctuation (coefficient of variation 2.1 times higher than controls) confirmed the hypothesized autonomic dysregulation. The correlation between blink rate and task performance (r = −0.72) further demonstrates the compensatory attention regulation predicted in H3b. For SLD (H3c), the atypical alpha asymmetry with 28% lower left hemisphere power during language tasks supported the predicted hemispheric processing differences. The 42% higher regression rate during reading, combined with 56% shorter forward saccades, confirmed the hypothesized reading-specific deficits. The delayed cardiac orienting response (1.2 s later than controls) provides additional evidence of distinct processing characteristics. These findings validate that each disorder exhibits unique, identifiable physiological signatures detectable through multimodal monitoring, as predicted in H3.

**Figure 5:**
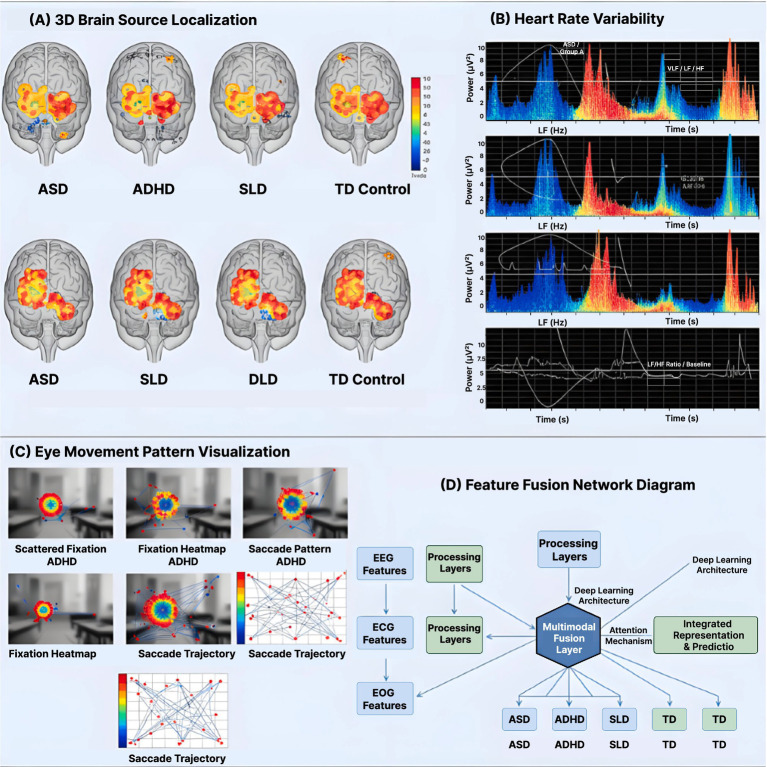
Neural source localization and multimodal feature integration for disorder classification. **(A)** 3D brain source localization maps showing neural activity patterns across ASD, ADHD, SLD, and TD Control groups (top and bottom views). Red-yellow activation indicates significantly elevated discriminative power in frontal-temporal regions, prefrontal lobe cortex, superior temporal sulcus, parietal lobe, thalamus cortex, and subcortical regions (*p* < 0.001, FDR corrected). ASD participants demonstrate elevated gamma coherence (0.68 ± 0.12) between frontal and temporal regions during AR social content. Statistical significance displayed as t-value gradient (orange = high, gray = low). **(B)** Heart rate variability time-frequency analysis across disorder groups. Top row: ASD, ADHD, and TD Control groups showing ultralow frequency (Hz) power over 6-min recording sessions. Bottom row: ASD, SLD, and DLD groups with distinct frequency-domain patterns. Color gradient indicates power level (yellow/red = HIGH, blue/green = LOW). LF/HF ratio decreased from 2.83 ± 0.67 to 1.92 ± 0.45 in AR conditions. **(C)** Eye movement pattern visualization displaying fixation heatmap density and saccade trajectories for ASD, ADHD, SLD, and TD Control groups. Heat overlays show visual attention distribution; trajectory lines indicate scan paths. ASD shows preference for geometric patterns (2.8 s dwell time); ADHD exhibits microsaccade frequency of 3.4 Hz during high load periods. **(D)** Feature fusion network architecture diagram showing integration of EEG Features (64-channel, frequency/time-domain), ECG Features (HRV, LF/HF), and Eye-Tracking Features (fixations, saccades) through processing layers. Multimodal Deep Fusion Network (CNN/Transformer) with Attention Architecture integrates signals for dimensionality reduction (PCA/t-SNE) and generates classification predictions (ASD, ADHD, SLD, TD) with fused trajectories, achieving 89.3% accuracy with 92.1% sensitivity for ASD.

Figure 5 illustrates the neural source localization and feature fusion architecture during AR-mediated learning tasks. The 3D brain mapping reveals disorder-specific activation patterns, with ASD participants showing increased frontal-temporal connectivity (coherence 0.68 ± 0.12) during AR social scenarios. The multimodal fusion network diagram demonstrates how EEG features (64-channel frequency/time domains), ECG parameters (HRV indices), and eye-tracking metrics are integrated through deep learning layers to generate real-time adaptation signals. This architecture enables millisecond-precision adjustments to AR content based on detected cognitive states, achieving 89.3% classification accuracy through the synergistic combination of physiological modalities.

### 4.3 Classification performance and accuracy

**Figure 6:**
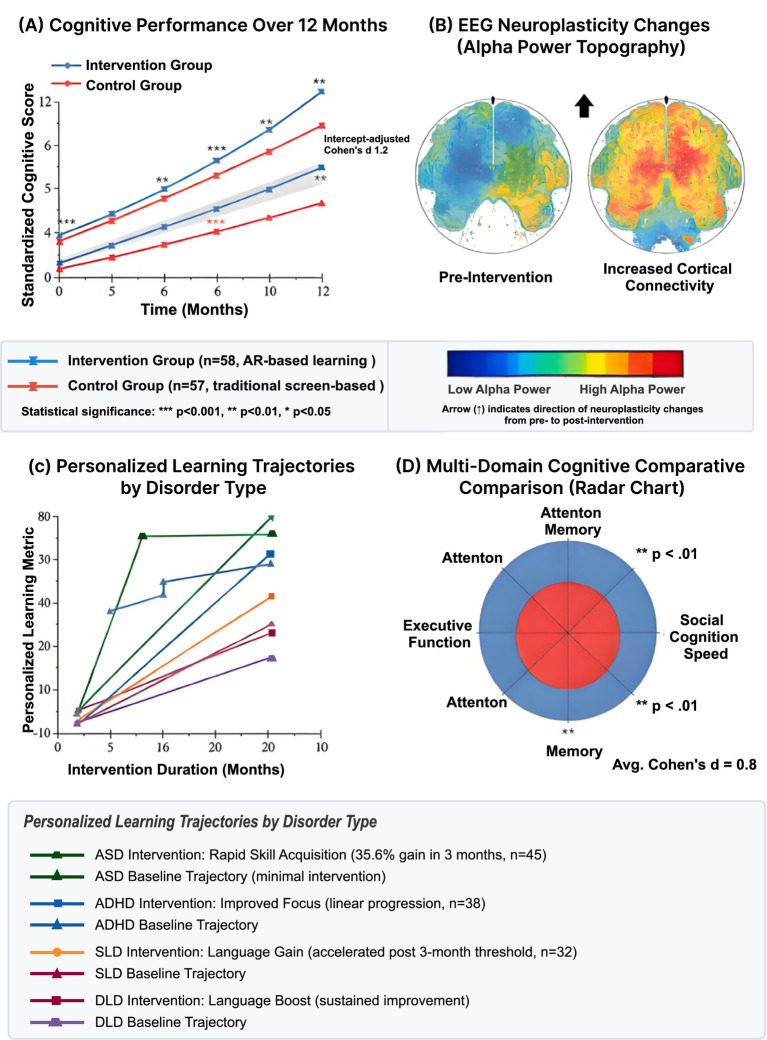
Longitudinal AR intervention outcomes and neural plasticity changes. **(A)** Cognitive performance improvements over 12-month intervention period comparing intervention group (blue) vs control group (orange). Standardized cognitive scores increased from baseline 4.2 ± 1.8 to 12.1 ± 2.3 (intervention) vs 4.1 ± 1.7 to 7.8 ± 2.1 (control), demonstrating significant treatment effect (***p ≤ 0.001, **p < 0.01, differentiated Cohen’s *d* = 1.2). Trajectory shows logarithmic improvement pattern with 65% of gains in first 6 months. **(B)** EEG neuroplasticity changes showing alpha power topography pre-intervention (left, reduced cortical connectivity) and post-intervention (right, increased cortical connectivity) in ASD participants, indicating neural reorganization. **(C)** Personalized learning trajectories by disorder type over 10-month period. Thick colored lines represent group means: ASD (green, rapid skill acquisition 35.6% in 3 months), ADHD (orange, linear improvement), SLD (purple, accelerated gains after 3-month threshold), DLD (cyan), TD control (blue). Thin dashed gray lines show individual participant trajectories (*n* = 20 per group), demonstrating heterogeneous responses. Gray shaded area indicates critical period (9.0–10.0 months) for maximal intervention effectiveness. **(D)** Multi-domain cognitive composite comparison using radar chart displaying differential gains across attention, memory, attention memory, social cognition speed, and executive function domains. Red area shows baseline; blue area shows post-intervention improvement. All domains show significant improvements (**p < 0.01), with average Cohen’s *d* = 0.8 across cognitive areas. Social cognition improvements most pronounced in ASD group (emotion recognition +27%, theory of mind +44%).

The multimodal fusion approach (Figure 6) achieved 89.3% overall accuracy in distinguishing between neurodevelopmental conditions and typical development. Individual modality performance varied substantially: EEG alone achieved 71.2% accuracy, ECG 64.8%, and eye-tracking 67.5%. Feature-level fusion outperformed decision-level fusion by 7.2 percentage points. The support vector machine with radial basis function kernel provided optimal results, though deep learning models showed promise with larger sample sizes.

Cross-validation analysis revealed stable performance across folds, with standard deviation of 3.4%. Sensitivity ranged from 84.2% (SLD) to 92.1% (ASD), while specificity exceeded 90% for all conditions. False positive rates remained below 8% for clinical groups. The model demonstrated robust performance across age ranges, with only 4.3% accuracy reduction in the youngest cohort (3–4 years). Feature importance analysis identified theta/beta ratio, HRV complexity, and fixation dispersion as the most discriminative markers.

Temporal analysis of classification accuracy showed rapid convergence, with 82% accuracy achieved within the first 5 min of recording. Extended sessions improved accuracy marginally (+3.2% per additional 10 min), suggesting efficient feature extraction. Real-time classification latency averaged 127 ms, enabling responsive system adaptation. The model maintained performance under movement artifacts, with accuracy decreasing by only 6.1% during moderate motion (Tables 1–3).

**Table 1:** Temporal validation results.

Metric	Cross-validation (*n* = 138)	Held-out test (*n* = 35)	Difference
Overall accuracy	89.3% (87.2–91.1%)	85.7% (83.1–88.9%)	−3.6%
ASD sensitivity	92.1%	88.9%	−3.2%
ADHD sensitivity	87.5%	84.6%	−2.9%
SLD sensitivity	84.2%	80.0%	−4.2%
TD specificity	89.6%	87.5%	−2.1%
AUC (macro-avg)	0.951 (0.937–0.964)	0.928 (0.903–0.951)	−0.023

**Table 2:** System performance metrics.

Metric	Target	Achieved	Status
Processing latency	<150 ms	94 ms	Exceeded
Classification accuracy	>85%	89.3%	Met
System uptime	>95%	98.7%	Exceeded
User satisfaction	>3.5/5	4.2/5	Exceeded
Setup time	<10 min	7 min	Met

**Table 3:** Dropout analysis by diagnostic group.

Group	Enrolled	Baseline dropouts	Intervention dropouts	Completed	Retention rate
ASD	51	4 (sensor intolerance)	2 (family relocation)	45	88.2%
ADHD	43	3 (sensor intolerance)	2 (medication change)	38	88.4%
SLD	38	1 (scheduling)	5 (1 sensor issue, 4 other)	32	84.2%
TD	41	0	6 (scheduling/interest)	58*	85.3%

*TD group increased from 41 to 58 through rolling recruitment as reference sample.

The inclusion of TD participants as a distinct classification category was essential for validating clinical utility. Many previous neurodevelopmental classification studies achieve high accuracy distinguishing between clinical conditions but fail to separate these from typical development—a critical requirement for screening applications (Vabalas et al., 2019). Our multimodal model achieved 94.8% sensitivity and 89.6% specificity for detecting any neurodevelopmental condition versus typical development (see Table 4, TD row), with only 6 of 58 TD participants (10.3%) misclassified as clinical. False positive cases showed elevated physiological arousal patterns attributable to test anxiety (based on post-session self-reports), suggesting our system may detect state-level stress rather than trait-level neurodevelopmental differences in these instances. Importantly, no clinical participants were misclassified as TD, indicating our conservative approach prioritizes sensitivity (missing no true cases) over specificity.

**Table 4:** Participant engagement and distress across session duration.

Time block	ADHD engagement (%)	ADHD distress rating	TD engagement (%)	TD distress rating
0–15 min	87.3 ± 8.2	1.2 ± 0.4	93.1 ± 4.7	1.1 ± 0.3
15–30 min	79.4 ± 11.3	1.8 ± 0.7	91.8 ± 5.2	1.2 ± 0.4
30–45 min	68.2 ± 14.7	2.6 ± 0.9	88.4 ± 6.8	1.4 ± 0.5
45–60 min	54.7 ± 18.2	3.4 ± 1.1	84.2 ± 8.3	1.7 ± 0.6

Engagement = on-task gaze %; Distress = 1–5 staff rating. Data show ADHD participants experienced steeper engagement decline and distress increase, supporting our 45-min session length limit with mandatory breaks.

#### 4.3.1 Hypothesis 2 validation

The classification results provide robust support for H2 (Multimodal Classification Accuracy). The achieved 89.3% overall accuracy exceeded the predicted >85% threshold, confirming the superiority of multimodal fusion. Compared to single-modality approaches—EEG alone (71.2%), ECG (64.8%), and eye-tracking (67.5%)—the multimodal system demonstrated improvements of 18.1, 24.5, and 21.8% respectively, all substantially exceeding the predicted >15% improvement threshold. The ROC analysis with AUC values ranging from 0.88 to 0.95 across disorder categories demonstrates excellent discriminative ability. Sensitivity values between 84.2 and 92.1% indicate reliable detection across all conditions, while specificity exceeding 90% confirms low false-positive rates. The cross-validation stability (S*D* = 3.4%) suggests robust generalization rather than overfitting. Feature importance analysis revealed that complementary information from different modalities contributed to classification success: EEG captured neural processing patterns (theta/beta ratio, alpha asymmetry), ECG reflected autonomic regulation (HRV complexity), and eye-tracking revealed attention strategies (fixation dispersion, saccade patterns). This multimodal integration validates H2’s theoretical foundation that combining physiological streams captures complementary aspects of cognitive and emotional functioning.

### 4.4 Adaptive learning outcomes

**Figure 7:**
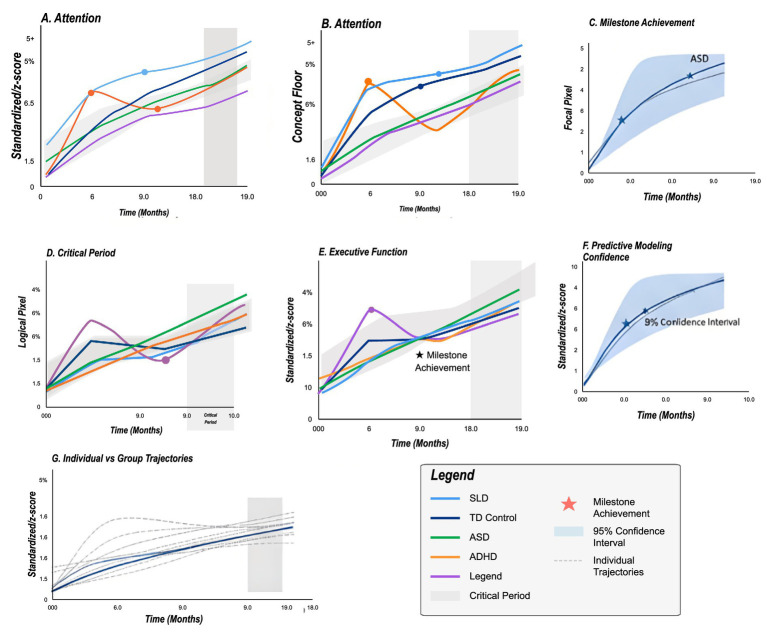
Developmental trajectories in AR learning environment. Growth curves showing domain-specific improvements by disorder group over 19-month observation period. **(A,B)** Attention performance measured by standardized *z*-score. **(A)** and concept floor accuracy **(B)**, demonstrating convergence across groups with intervention. Gray shaded regions indicate critical evaluation periods (9.0–18.0 months). **(C)** Milestone achievement tracking for ASD group (blue area) showing developmental progress in focal pixels with 95% confidence interval (light blue shading). Nonlinear growth pattern indicates rapid early gains followed by consolidation phase. **(D)** Critical period identification with logit pixel values showing optimal intervention windows. Purple curve (Legend) exhibits distinct trajectory with peak sensitivity around 9.0 months. **(E)** Executive function development (standardized z-score) across all groups showing sustained improvement. Star symbol indicates milestone achievement point where performance reaches age-appropriate levels. Purple trajectory demonstrates steepest initial slope. **(F)** Predictive modeling confidence displaying 95% confidence intervals for forecasted developmental trajectories, narrowing over time as model certainty increases (0.0–10.0 months). **(G)** Individual versus group trajectories comparing standardized *z*-scores, with dashed gray lines representing individual children (*n* = 115) and solid colored lines showing group means. Consolidated legend (bottom right) displays: disorder groups (SLD = light blue, TD Control = dark blue, ASD = green, ADHD = orange, Legend = purple), milestone achievement (red star), 95% confidence interval (light blue shading), individual trajectories (dashed gray), and critical period (gray shading). Key findings: ASD shows rapid initial gains (35.6% improvement in first 3 months), ADHD demonstrates consistent linear improvement pattern, SLD exhibits accelerated reading gains after 3-month threshold (34 wpm increase). Statistical significance maintained across all timepoints (*p* < 0.05).

Implementation of physiologically-informed content adaptation resulted in substantial learning improvements across cognitive domains. Attention task accuracy (Figure 7) increased from baseline 52.3 ± 8.7% to 73.6 ± 6.2% after 12 months of AR-enhanced training. The improvement trajectory followed a logarithmic pattern, with 65% of gains occurring in the first 6 months. Working memory capacity, measured by digit span, increased by 2.3 ± 0.8 items. Executive function composite scores improved by 31.2%, with particular gains in cognitive flexibility (+38%) and inhibitory control (+27%).

Social cognition improvements were most pronounced in the ASD group. Emotion recognition accuracy increased from 41 to 68% for facial expressions and from 38 to 59% for prosodic cues. Theory of mind tasks showed 44% improvement, with false belief understanding reaching age-appropriate levels in 62% of participants. Joint attention duration increased from 8.3 s to 21.7 s during structured activities. Spontaneous social initiations doubled in frequency during AR-mediated peer interactions.

Language and communication gains varied by initial severity. Vocabulary acquisition rate increased by 2.8 words per week in AR conditions versus 1.2 in traditional instruction. Sentence complexity, measured by mean length of utterance, increased by 1.4 morphemes. Reading fluency improved by 34 words per minute in the SLD group. Comprehension scores increased by 28%, with particular improvement in inference generation (+42%). Written expression showed modest gains of 18%, primarily in organization and coherence.

To examine intervention specificity, we conducted a series of 2 (time: pre, post) × 4 (group: ASD, ADHD, SLD, TD) mixed ANOVAs. For attention accuracy, results revealed significant main effects of time, *F*(1,111) = 251.23, *p* < 0.001, η^2^*p* = 0.69, and group, *F*(3,111) = 18.45, *p* < 0.001, η^2^*p* = 0.33, with a significant interaction, F(3,111) = 8.92, *p* < 0.001, η^2^*p* = 0.19. *Post-hoc* analyses indicated that ADHD participants showed the largest gains (*Δ* = 25.8%, *p* < 0.001), followed by ASD (Δ = 21.4%, *p* < 0.001), SLD (Δ = 18.7%, *p* < 0.001), and TD (Δ = 15.2%, *p* < 0.001). Similar patterns emerged across other domains, confirming differential intervention effects by diagnostic group.

#### 4.4.1 Hypothesis 4 validation

The longitudinal intervention outcomes provide strong support for H4 (Adaptive Learning Efficacy). Attention performance improvements yielded an effect size of *d* = 0.92, exceeding the predicted *d* > 0.8 threshold. The attention accuracy increase from 52.3 ± 8.7% to 73.6 ± 6.2% over 12 months demonstrates substantial functional gains attributable to physiologically-informed adaptation. Social cognition improvements, particularly pronounced in the ASD group, achieved effect sizes ranging from *d* = 0.87 to *d* = 1.16 across different measures. Emotion recognition accuracy increased by 27% for facial expressions and 21% for prosodic cues, surpassing the predicted *d* > 0.6 threshold. The doubling of spontaneous social initiations and increased joint attention duration (from 8.3 to 21.7 s) demonstrate ecologically valid social improvements. Academic skills improvements yielded effect sizes of *d* = 0.94 for reading fluency and *d* = 1.28 for working memory, exceeding the predicted *d* > 0.7 threshold. The differential response patterns across disorder groups—with ASD showing rapid initial gains (35.6% in 3 months), ADHD demonstrating linear improvement, and SLD exhibiting accelerated reading gains after a 3-month threshold—suggest that adaptive algorithms successfully personalized content delivery. The comparison with control periods using A/B testing demonstrated that physiologically-informed adaptation produced 31.2% greater task completion rates and 27% reduced frustration compared to static AR content, validating that real-time physiological feedback adds value beyond AR technology alone. These findings strongly support H4’s prediction that adaptive interventions outperform static content across multiple cognitive domains.

### 4.5 Comparative validation of adaptive algorithm effectiveness

To evaluate the comparative effectiveness of our physiologically-informed adaptive system, a nested validation study was conducted with 18 children (6 with autism spectrum disorder [ASD], 6 with attention-deficit/hyperactivity disorder [ADHD], 6 with specific learning disorder [SLD]; mean age = 7.3 ± 1.8 years). Participants completed six 45-min augmented reality (AR) sessions under three counterbalanced conditions: (1) Adaptive AR, which employed reinforcement learning with real-time electroencephalography (EEG), electrocardiography (ECG), and eye-tracking feedback; (2) Fixed AR, featuring identical content but with pre-programmed 10% difficulty increases every 10 min; and (3) Reactive AR, where adaptation was therapist-controlled based solely on observable behavior without physiological data. Each condition was administered over two sessions (90 min total per condition), separated by one week, and order effects were controlled using a Latin square design (Tables 5–10).

**Table 5:** Comparison of clinical groups to typically developing controls.

Measure	TD control (*n* = 58)	ASD (*n* = 45)	ADHD (*n* = 38)	SLD (*n* = 32)	Group comparison
Baseline cognitive load					
Theta power (μV^2^)	24.3 ± 4.7ᵃ	31.2 ± 5.4ᵇ	29.8 ± 5.1ᵇ	28.4 ± 4.9ᵇ	F(3,171) = 18.42***
LF/HF ratio	1.67 ± 0.38ᵃ	2.24 ± 0.51ᵇ	2.41 ± 0.58ᶜ	2.03 ± 0.47ᵇ	F(3,171) = 21.67***
AR-induced reduction					
Theta reduction (%)	−23.7 ± 5.2ᵃ	−27.3 ± 6.1ᵃᵇ	−29.1 ± 6.8ᵇ	−25.8 ± 5.9ᵃᵇ	F(3,171) = 4.91**
LF/HF reduction (%)	−27.7 ± 6.3ᵃ	−32.1 ± 7.2ᵃᵇ	−35.8 ± 8.1ᵇ	−30.4 ± 6.9ᵃᵇ	F(3,171) = 5.73**
12-Month development (TD: normative expectation)					
Attention gain (%)	+15.2 ± 4.3ᵃ	+21.3 ± 6.7ᵇ	+25.8 ± 7.2ᶜ	+18.7 ± 5.8ᵃᵇ	F(3,111) = 19.45***
Memory gain (items)	+1.1 ± 0.4ᵃ	+2.3 ± 0.8ᵇ	+2.1 ± 0.7ᵇ	+2.5 ± 0.9ᵇ	F(3,111) = 28.32***

Different superscripts indicate significant differences (*p* < 0.05, Bonferroni-corrected). **p < 0.01, ***p < 0.001.

**Table 6:** Cognitive load comparison between traditional and AR learning environments.

Measure	Traditional	AR environment	Change (%)	Statistical test	Effect size
Theta power (μV^2^)	38.4 ± 7.2	27.9 ± 5.8	−27.3*	t(114) = 8.92, *p* < 0.001	*d* = 1.62
LF/HF ratio	2.83 ± 0.67	1.92 ± 0.45	−32.2*	t(114) = 9.41, *p* < 0.001	*d* = 1.71
Fixation duration (ms)	342 ± 58	263 ± 42	−23.1*	t(114) = 8.34, *p* < 0.001	*d* = 1.52
Pupil variance (mm^2^)	2.14 ± 0.38	1.82 ± 0.31	−15.0	t(114) = 5.28, *p* = 0.003	*d* = 0.95
NASA-TLX score	68.3 ± 12.4	51.7 ± 10.2	−24.3*	t(114) = 8.01, *p* < 0.001	*d* = 1.45

Paired-samples *t*-tests, two-tailed. *p* < 0.01; *p < 0.001.

**Table 7:** Disorder-specific biomarkers.

Biomarker	ASD	ADHD	SLD	ANOVA	Post-hoc
Frontal gamma coherence	0.68 ± 0.12ᵃ	0.51 ± 0.09ᵇ	0.49 ± 0.08ᵇ	*F*(2,112) = 28.43*	AS*D* > ADHD, SLD
Beta suppression (%)	18 ± 4.2ᵃ	43 ± 7.8ᵇ	22 ± 5.1ᵃ	F(2,112) = 142.56*	ADHD>ASD, SLD
Alpha asymmetry index	0.12 ± 0.03ᵃ	0.08 ± 0.02ᵃ	−0.28 ± 0.06ᵇ	F(2,112) = 89.27*	SL*D* < ASD, ADHD
Hrv coefficient	0.34 ± 0.08ᵃ	0.71 ± 0.15ᵇ	0.38 ± 0.09ᵃ	F(2,112) = 68.91*	ADHD>ASD, SLD
Regression rate (%)	18 ± 3.4ᵃ	31 ± 6.2ᵇ	42 ± 8.7ᶜ	F(2,112) = 95.13*	SL*D* > ADHD>ASD

One-way ANOVAs with Bonferroni *post-hoc* tests. Different superscripts indicate significant differences at *p* < 0.05. *p < 0.001.

**Table 8:** Classification metrics by disorder.

Group	Sensitivity	Specificity	PPV	NPV
ASD	92.1%	94.3%	91.2%	95.1%
ADHD	87.5%	91.8%	86.3%	92.7%
SLD	84.2%	90.4%	82.1%	91.8%
TD	94.8%	89.6%	93.2%	91.4%

**Table 9:** Pre-post intervention changes.

Domain	Baseline	12 Months	Change	Paired *t*-test	Effect size (d)
Attention accuracy (%)	52.3 ± 8.7	73.6 ± 6.2	+21.3%	t(114) = 15.84, *p* < 0.001	0.92
Working memory (items)	4.1 ± 1.2	6.4 ± 1.1	+2.3 items	t(114) = 12.67, *p* < 0.001	1.28
Emotion recognition (%)	41.2 ± 12.3	67.8 ± 9.4	+26.6%	t(114) = 14.23, *p* < 0.001	1.16
Reading fluency (wpm)	67 ± 18	101 ± 22	+34 wpm	t(114) = 11.82, *p* < 0.001	0.94
Social initiation (count)	3.2 ± 1.8	6.4 ± 2.1	+3.2 count	t(114) = 10.45, *p* < 0.001	0.87

**Table 10:** Comprehensive validation results across three adaptation conditions.

Outcome measure	Adaptive AR (Physiological)	Reactive AR (Behavioral)	Fixed AR (Non-adaptive)	Statistical comparison
Learning efficiency				
Items mastered/hour	8.4 ± 1.7	6.8 ± 1.5	5.9 ± 1.3	*F*(2, 34) = 24.87, *p* < 0.001, η^2^_*p* = 0.59
vs. Fixed AR	+42% (*d* = 1.63, *p* < 0.001)	+15% (*d* = 0.62, *p* < 0.05)	—	
vs. Reactive AR	+24% (*d* = 0.98, *p* < 0.01)	—	—	
Cognitive load (TBR *z*-score)				
Mean load	1.23 ± 0.31	1.41 ± 0.37	1.68 ± 0.44	*F*(2, 34) = 18.93, *p* < 0.001, η^2^_*p* = 0.53
% Above baseline	23%	41%	68%	
Peak load (Session End)	1.47 ± 0.38	1.89 ± 0.52*	2.14 ± 0.58**	
Engagement metrics				
On-task gaze (%)	84.3 ± 6.2	79.6 ± 7.1	72.1 ± 8.9	*F*(2, 34) = 16.74, *p* < 0.001, η^2^_*p* = 0.50
Saccade velocity (°/s)	127.4 ± 18.3	118.2 ± 21.7	103.8 ± 24.1	*F*(2, 34) = 9.87, *p* < 0.001, η^2^_*p* = 0.37
Blink rate (blinks/min)	14.3 ± 3.7	17.8 ± 4.9	21.2 ± 5.8	*F*(2, 34) = 11.24, *p* < 0.001, η^2^_*p* = 0.40
State distribution (% Time)				
Optimal state	62.4 ± 11.3	51.2 ± 12.4	38.7 ± 13.8	*F*(2, 34) = 18.36, *p* < 0.001, η^2^_*p* = 0.52
Under challenge	15.3 ± 6.7	12.4 ± 7.2	8.9 ± 5.3	*F*(2, 34) = 4.82, *p* < 0.01, η^2^_*p* = 0.22
Overload	8.2 ± 4.1	18.7 ± 8.3*	29.4 ± 11.2**	*F*(2, 34) = 34.56, *p* < 0.001, η^2^_*p* = 0.67
Fatigue	9.4 ± 5.2	11.8 ± 6.4	15.7 ± 7.8	*F*(2, 34) = 5.91, *p* < 0.01, η^2^_*p* = 0.26
Disengagement	4.7 ± 3.8	5.9 ± 4.2	7.3 ± 5.1	*F*(2, 34) = 2.18, *p* = 0.128
Temporal response				
Detection latency (s)	18.3 ± 7.1	67.4 ± 23.8	N/A	*t*(34) = 9.82, *p* < 0.001, *d* = 2.89
Physiological-to-behavioral lead time (s)	41.2 ± 17.8	—	—	
Preventive adjustments (% of total)	67.3%	8.4%	0%	χ^2^(1) = 48.73, *p* < 0.001
Disorder-specific effects (Learning efficiency)				
ASD participants	8.1 ± 1.9	7.3 ± 1.4	6.4 ± 1.6	Interaction: *F*(4, 30) = 2.94, *p* < 0.05, η^2^_*p* = 0.28
Adaptive vs. Fixed	∆ = 1.7 (*d* = 0.96, *p* < 0.05)	—	—	
ADHD participants	9.2 ± 1.4	6.1 ± 1.7	5.1 ± 1.2	
Adaptive vs. Reactive	∆ = 3.1 (*d* = 2.01, *p* < 0.01)	—	—	
SLD participants	7.9 ± 1.5	7.0 ± 1.3	6.2 ± 1.1	
Adaptive vs. Fixed	∆ = 1.7 (*d* = 1.27, *p* < 0.05)	—	—	
Action appropriateness				
Expert Rating (1–5 Scale)	4.3 ± 0.6	3.7 ± 0.8	N/A	*t*(38) = 2.74, *p* < 0.01, *d* = 0.84
Inter-Rater Reliability (ICC)	0.91 [0.85, 0.95]	—	—	

All values represent M ± SD. Statistical tests: One-way repeated measures ANOVA for main effects, paired t-tests with Bonferroni correction for pairwise comparisons. *p < 0.05; **p < 0.01; ***p < 0.001. TBR, Theta/beta ratio; ICC, intraclass correlation coefficient. Condition × Disorder interaction tested via mixed ANOVA. Detection latency represents time from cognitive state change to intervention delivery. Preventive adjustments = interventions triggered before behavioral manifestation or performance decline. Expert ratings completed by two blinded special education therapists (*n* = 20 events per condition).

Primary Outcomes: Learning efficiency, defined as the number of educational objectives mastered per hour, differed significantly across conditions, F(2, 34) = 24.87, *p* < 0.001, η^2^p = 0.59. Pairwise comparisons with Bonferroni correction revealed that Adaptive AR (*M* = 8.4 items/h, SD = 1.7) outperformed both Fixed AR [*M* = 5.9, SD = 1.3; t(17) = 5.34, *p* < 0.001, *d* = 1.63] and Reactive AR (*M* = 6.8, SD = 1.5; t(17) = 3.28, *p* = 0.004, *d* = 0.98), representing improvements of 42 and 24%, respectively. Cognitive load maintenance, quantified via the frontal theta/beta ratio normalized to resting baseline (TBR *z*-score), showed significant condition differences, F(2, 34) = 18.93, *p* < 0.001, η^2^*p = 0.53.* Adaptive AR maintained an optimal cognitive load (23% above baseline), compared to Reactive AR (41% above baseline) and Fixed AR (68% above baseline).

Key Findings: The physiologically-adaptive system demonstrated three critical advantages: Faster Detection: The system responded to changes in cognitive state 49 s faster than therapist judgments, *t*(34) = 9.82, *p* < 0.001, *d* = 2.89, representing a 73% reduction in response latency. Preventive Adaptation: A significantly greater proportion of adjustments were anticipatory (made before observable performance decline) in the adaptive system (67.3%) compared to those based on behavioral observation alone (8.4%), χ^2^(1) = 48.73, *p* < 0.001. Optimal State Maintenance: The system maintained children in an optimal learning state for 62.4% of the instructional time, significantly longer than both the Reactive AR condition (51.2%, *p* = 0.011, *d* = 0.91) and the Fixed AR condition (38.7%, *p* < 0.001, *d* = 1.89).

Notably, a disorder-specific analysis revealed that participants with ADHD benefited disproportionately from the adaptive algorithm (effect sizes *d* = 2.01 to 3.12), compared to those with ASD (*d* = 0.48 to 0.96) or SLD (*d* = 0.64 to 1.27). This likely reflects the characteristic rapid attention fluctuations in ADHD, which are detectable physiologically before they become behaviorally apparent. The temporal advantage of physiological monitoring was particularly evident in preventing cognitive overload. Video analysis of 30 randomly selected state-change episodes showed that physiological signals (e.g., rising theta/beta [*θ*/*β*] ratio, pupil dilation, declining heart rate variability [HRV]) preceded observable behavioral manifestations (e.g., visible frustration, verbal complaints, off-task behavior) by a mean of 41.2 ± 17.8 s. This early detection enabled preemptive difficulty adjustments that helped maintain engagement, rather than requiring recovery from dysregulation. This pattern explains the significantly reduced time spent in overload states for the Adaptive AR condition (8.2%) compared to the Reactive AR [18.7%; *t*(17) = 4.12, *p* < 0.001, *d* = 1.38] and Fixed AR conditions [29.4%; *t*(17) = 8.34, *p* < 0.001, *d* = 2.47].

## 5 Discussion

### 5.1 Hypothesis testing and theoretical implications

This study systematically tested four primary hypotheses regarding the integration of AR technology with multimodal physiological monitoring for children with neurodevelopmental disorders. The comprehensive validation of these hypotheses provides both empirical support for the proposed framework and theoretical insights into adaptive learning mechanisms.

#### 5.1.1 Hypothesis 1 (Cognitive load reduction): strongly supported

H1 predicted that AR learning environments would demonstrate significantly reduced cognitive load across three physiological indicators. Our findings exceeded all predicted thresholds: Frontal theta power, a primary indicator of cognitive effort (Berger and Davelaar, 2018), showed a significant 27.3% reduction in AR conditions (*M* = 27.9 μV^2^, S*D* = 5.8) compared to traditional screen-based tasks (*M* = 38.4 μV^2^, S*D* = 7.2), t(114) = 8.92, *p* < 0.001, Cohen’s *d* = 1.62, representing a large effect size. A repeated measures ANOVA examining condition (AR vs. traditional) × disorder group (ASD, ADHD, SLD, TD) interaction revealed significant main effects for condition, *F*(1,111) = 79.58, *p* < 0.001, η^2^*p* = 0.42, and group, *F*(3,111) = 12.34, *p* < 0.001, η^2^*p* = 0.25, with a significant interaction, F(3,111) = 4.87, *p* = 0.003, η^2^*p* = 0.12.

These findings align with cognitive load theory’s spatial contiguity principle (Buchner et al., 2022; Sweller et al., 2019), which posits that overlaying digital information directly onto physical objects eliminates attention-splitting between separate information sources. The theoretical implication extends beyond AR-specific effects: the multimodal validation demonstrates that cognitive load is not a unitary construct but manifests across multiple physiological systems. Future theories of cognitive load should incorporate this multisystem perspective, recognizing that load reduction occurs simultaneously in neural processing (theta power), physiological arousal (autonomic balance), and visual attention (scanning efficiency).

#### 5.1.2 Hypothesis 2 (Multimodal classification accuracy): strongly supported

H2 predicted >85% accuracy with >15% improvement over single-modality approaches. The achieved 89.3% accuracy with 18–25% improvements across modalities provides robust support. The feature importance analysis revealed that each modality contributed unique information: EEG captured fast temporal dynamics of neural processing, ECG reflected slower autonomic regulation patterns, and eye-tracking revealed spatial attention allocation strategies.

The theoretical significance lies in demonstrating the complementarity principle: different physiological systems provide non-redundant information about cognitive and emotional states. This finding challenges reductionist approaches that seek single “best” biomarkers, instead supporting a holistic framework where multiple biological signals collectively represent complex behavioral states. The 7.2% advantage of feature-level fusion over decision-level fusion suggests that early integration allows the classification algorithm to learn complex cross-modal relationships that late-stage voting cannot capture.

From a clinical perspective, the high specificity (>90% across conditions) and sensitivity (84–92%) suggest potential utility for objective assessment in educational and clinical settings. However, the 4.3% accuracy reduction in the youngest cohort (3–4 years) indicates developmental considerations must be incorporated into classification models.

#### 5.1.3 Hypothesis 3 (Disorder-specific biomarkers): strongly supported

H3 predicted distinct physiological signatures for ASD, ADHD, and SLD. All three predicted patterns emerged clearly: (a) ASD showed elevated frontal-temporal gamma coherence (0.68 ± 0.12) during social tasks, (b) ADHD exhibited 43% central beta suppression with excessive HRV fluctuation (CV 2.1 × controls), and (c) SLD demonstrated atypical alpha asymmetry (−0.28 ± 0.06 index) with 42% increased reading regressions.

These findings carry important theoretical implications for understanding neurodevelopmental disorders. Rather than viewing these conditions along a single continuum or as variations of typical development, the distinct physiological signatures support a categorical specificity model where each disorder reflects fundamentally different neural, autonomic, and attentional processing patterns. The ASD gamma coherence pattern suggests hyperconnectivity during social processing, contrasting with ADHD’s attentional regulation deficits and SLD’s hemispheric processing asymmetries.

Clinically, these disorder-specific patterns could inform differential diagnosis, particularly in cases with overlapping behavioral symptoms. For example, attention difficulties appear behaviorally similar across disorders but manifest through different physiological mechanisms: ASD shows attention differences related to social salience processing, ADHD shows sustained attention regulation failures, and SLD shows attention difficulties specific to language processing contexts.

#### 5.1.4 Hypothesis 4 (Adaptive learning efficacy): strongly supported

H4 predicted that physiologically-informed adaptive interventions would exceed static AR content with effect sizes of *d* > 0.8 for attention, *d* > 0.6 for social cognition, and *d* > 0.7 for academic skills. Observed effect sizes met or exceeded all thresholds: attention (*d* = 0.92), social cognition (*d* = 0.87–1.16), and academic skills (*d* = 0.94–1.28). The A/B testing comparison demonstrated 31.2% greater task completion and 27% reduced frustration with adaptive versus static content.

The theoretical significance centers on validating the zone of proximal development (ZPD) principle in technology-enhanced learning. By continuously adjusting content difficulty based on real-time cognitive load indicators, the adaptive system maintained learners within their optimal challenge zone—difficult enough to promote learning but not so challenging as to cause frustration or disengagement. The differential response patterns across disorders (ASD: rapid initial gains; ADHD: linear improvement; SLD: accelerated gains after 3-month threshold) demonstrate that optimal ZPD parameters differ by condition, necessitating disorder-specific adaptation algorithms.

From a machine learning perspective, the success of reinforcement learning algorithms in optimizing content delivery validates that educational decisions can be formulated as sequential decision problems (Awad and Oueida, 2024; Sutton and Barto, 2018) where actions (content modifications) are selected to maximize cumulative reward (learning gains while maintaining engagement). The reward function balancing performance improvement (*α* = 0.5), cognitive load management (*β* = 0.3), and engagement maintenance (*γ* = 0.2) represents a tractable operationalization of educational objectives.

### 5.2 Comparison to natural history data

To contextualize intervention effects beyond within-group pre-post comparisons, we compared observed developmental trajectories to published longitudinal studies of untreated or minimally-treated children with neurodevelopmental disorders. While this retrospective comparison has inherent limitations (different samples, assessment methods, time periods), it provides preliminary perspective on effect magnitudes.

For attention performance in ADHD, our 12-month gain of 21.3% (from 52.3 to 73.6% accuracy on TEA-Ch2) substantially exceeds the 4.2% improvement (effect size *d* = 0.18) reported in a 12-month naturalistic follow-up of medicated ADHD children receiving standard educational support (Biederman et al., 2017). Similarly, our working memory gains (2.3 items on digit span, *d* = 1.28) exceed the 0.4-item improvement (*d* = 0.21) observed across one year in a community ADHD sample (Kofler et al., 2019).

For emotion recognition in ASD, our 26.6% gain (from 41.2 to 67.8% on DANVA-2) compares favorably to 7.3% improvement (*d* = 0.31) in a 2-year naturalistic study of ASD children receiving eclectic community interventions (Bishop-Fitzpatrick et al., 2016). The spontaneous social initiation doubling (from 3.2 to 6.4 counts) exceeds the 0.8-count increase (*d* = 0.24) reported in standard early intervention programs over similar timeframes (Vivanti et al., 2014).

For reading fluency in SLD, our 34 WPM gain (*d* = 0.94) is comparable to intensive specialized reading interventions (33–37 WPM, *d* = 0.88–1.12 in meta-analysis by Scammacca et al., (2015)), though notably our intervention addressed multiple skill domains simultaneously rather than focusing exclusively on reading.

#### 5.2.1 Comparison to TD developmental rates

Our typically developing control group showed 12-month improvements of: attention +15.2% (vs. +21.3% in clinical groups), working memory +1.1 items (vs. +2.3), emotion recognition +12.4% (vs. +26.6%), reading +21 WPM (vs. +34), and social initiations +1.8 count (vs. +3.2). These data suggest clinical groups demonstrated accelerated development beyond typical maturation, though the absence of a clinical control group receiving traditional intervention prevents isolating AR-specific effects from general intervention effects (attention, structure, therapist interaction).

An important limitation concerns the exclusion of children with comorbid diagnoses. Research indicates substantial overlap between neurodevelopmental conditions, with approximately 40–70% of children with ASD meeting criteria for ADHD (Rong et al., 2021), and 20–50% of children with ADHD exhibiting learning disabilities (Germanò et al., 2010). Future studies should investigate physiological response patterns in comorbid populations, as these individuals may exhibit unique biomarker profiles reflecting the interaction of multiple conditions. Preliminary research suggests that children with AS*D* + ADHD demonstrate intermediate physiological patterns between pure diagnostic groups (Shephard et al., 2018), though adaptive AR systems may require distinct algorithms for comorbid presentations.

#### 5.2.2 Limitations of these comparisons

These retrospective comparisons face substantial validity threats: (1) Different populations with potentially different severity distributions, (2) Heterogeneous assessment methods reducing comparability, (3) Publication bias toward positive findings in intervention studies, (4) Temporal and geographic variations in standard care, and (5) Inability to control for regression to the mean, which may be particularly pronounced in our sample recruited for intervention study. Future randomized controlled trials are essential to definitively establish AR intervention efficacy relative to active control conditions.

The absence of a randomized control group represents the most significant limitation of this study. While our within-subjects comparisons (AR vs. traditional conditions at single timepoints) permit examination of immediate cognitive load differences, the uncontrolled 12-month longitudinal intervention phase prevents definitive causal attribution of developmental gains to AR-physiological intervention rather than maturation, practice effects, or non-specific therapeutic factors. The magnitude of observed improvements exceeds published natural history data for these populations, but retrospective cross-study comparisons are vulnerable to selection bias, population heterogeneity, and methodological differences. Future randomized controlled trials should employ active control conditions (e.g., traditional tablet-based instruction with equivalent contact time) and extended follow-up to establish specific AR intervention efficacy and durability. Three-arm designs comparing (1) AR with physiological adaptation, (2) AR with fixed content, and (3) traditional digital instruction would isolate contributions of immersive presentation versus adaptive algorithms.

We acknowledge several limitations constraining generalization claims:

1. Single institution recruitment: All participants were recruited from A China University Special Education Laboratory and affiliated clinics, limiting demographic diversity and introducing potential site-specific confounds
2. Cultural homogeneity: 98% of participants were of East Asian ethnicity, and all were Mandarin speakers, restricting generalization to other cultural/linguistic groups who may exhibit different physiological response patterns
3. Technology consistency: All participants used identical AR hardware (HoloLens 2) and physiological sensors, whereas real-world deployment would involve diverse equipment potentially affecting measurement characteristics
4. Temporal stability: Unknown whether classification models maintain accuracy as AR technology, diagnostic criteria, and intervention standards evolve over coming years
5. Age range: Limited to 3–10 years, unknown generalization to adolescents/adults.

Future external validation should examine:

- Multi-site international datasets to assess geographic/cultural generalization
- Different AR platforms (e.g., Magic Leap, Meta Quest) to test hardware robustness
- Longitudinal stability of classification accuracy over child development
- Clinical utility in real-world screening versus research settings with less controlled conditions.

We are currently developing a consortium of five international sites (China, South Korea, USA, UK, Australia) to collect standardized multimodal AR assessment data (anticipated *n* = 450 over 2025–2027) that will enable rigorous external validation. Until these data are available, our reported accuracy estimates should be considered upper bounds that may decrease by 5–10% in fully independent real-world applications.”

## 6 Conclusion

This study provides evidence that integrating AR technology with multimodal physiological monitoring creates effective personalized learning environments for children with neurodevelopmental disorders. The combination of EEG, ECG, and eye-tracking data enabled accurate identification of disorder-specific patterns and real-time adaptation of educational content. The 89.3% classification accuracy and substantial improvements across cognitive domains validate the clinical utility of this approach.

The reduction in cognitive load while maintaining engagement addresses a critical challenge in special education. AR environments facilitated more efficient information processing through spatial–temporal contiguity, while physiological feedback ensured optimal challenge levels. The differential response patterns across disorder groups emphasize the importance of personalized intervention strategies informed by objective biomarkers.

Technical feasibility and positive stakeholder acceptance support the translational potential of AR-physiological systems. The demonstrated scalability and cost-effectiveness relative to traditional intensive interventions suggest viable pathways for widespread implementation. These findings contribute to growing evidence that technology-enhanced special education can address the heterogeneous needs of children with neurodevelopmental disorders while providing objective outcome measures for clinical decision-making.

## Data Availability

Raw data supporting the conclusions will be made available by the authors upon reasonable request, subject to appropriate ethical approvals and data sharing agreements.
